# Targeting mitochondrial tyrosyl-tRNA synthetase YARS2 suppresses colorectal cancer progression

**DOI:** 10.1080/15384047.2022.2127603

**Published:** 2022-09-25

**Authors:** Qingxia Fang, Jingyang Lin, Liang Gao, Ruolang Pan, Xiaochun Zheng

**Affiliations:** a Center for Clinical Pharmacy, Cancer Center, Department of Pharmacy, Zhejiang Provincial People’s Hospital (Affiliated People’s Hospital, Hangzhou Medical College), Hangzhou 310014, China; b Heart Center, Department of Cardiovascular Medicine, Zhejiang Provincial People’s Hospital (Affiliated People’s Hospital, Hangzhou Medical College), Hangzhou 310014, China; c Cancer Center, Department of Medical Oncology, Zhejiang Provincial People’s Hospital (Affiliated People’s Hospital, Hangzhou Medical College), Hangzhou 310014, China; d Key Laboratory of Cell-Based Drug and Applied Technology Development in Zhejiang Province, Institute for Cell-Based Drug Development of Zhejiang Province, Hangzhou, China

**Keywords:** YARS2, tyrosyl-tRNA, mitochondrial, colorectal cancer, reactive oxygen

## Abstract

Defects in tRNA expressions and modifications had been linked to various types of tumorigenesis and progression in recent studies, including colorectal cancer. In the present study, we evaluated transcript levels of mitochondrial tyrosyl-tRNA synthetase YARS2 in both colorectal cancer tissues and normal colorectal tissues using qRT-PCR. The results revealed that the mRNA expression level of YARS2 in colorectal cancer tissues was significantly higher than those in normal intestinal tissues. Knockdown of YARS2 in human colon cancer cell-line SW620 leads to significant inhibition of cell proliferation and migration. The steady-state level of tRNATyr, OCR, and ATP synthesis were decreased in the YARS2 knockdown cells. Moreover, our data indicated that inhibition of YARS2 is associated with increased reactive oxygen species levels which sensitize these cells to 5-FU treatment. In conclusion, our study revealed that targeting YARS2 could inhibit colorectal cancer progression. Thus, YARS2 might be a carcinogenesis candidate gene and can serve as a potential target for clinical therapy.

## Introduction

Colorectal cancer (CRC) is the third most common malignancy and the second deadly cancer worldwide,^1–4^ which induced nearly 2 million new cases and about 1 million deaths in the past year 2020 according to the World Health Organization database. Both the incidence and mortality have been increased in China during the last two decades.^5–7^ The reasons underlying this trend could be multifactorial, including genetic influences, changes in environmental, and also lifestyle exposures.^8,9^ A vast array of mutagens, mutations, as well as differentially expressed genes (DEGs), have been explored in CRC samples or cell lines by different groups of researchers.^9–11^ Despite the advances in cytotoxic and targeted therapy, surgery remains the primary choice of CRCs treatment. Thus, there is an urgent need to explore the further underlying mechanism of CRC progression and develop new therapeutic agents for the prevention and treatment of this heterogeneous disease.

Metabolic remodeling has been linked to various types of malignancies.^12–14^ It is now well accepted that the microenvironment changes during the process of tumor progression, and these transformed cells experienced metabolic adaptions as a response to these alternations.^15,16^ Chekkulayev et al. showed that CRC cells display upregulated oxidative phosphorylation (OXPHOS) compared with healthy colon tissues.^12^ Sun et al. also reported altered mitochondrial DNA (mtDNA) copy numbers on the progression of CRC.^17^ Wei et al. showed that metabolic targeting Hypoxia-inducible factor (HIF) −1a overcame chemoresistance and enhanced the anti-CRC treatment of oxaliplatin.^18^ However, the effects of altering metabolic on CRC cell progression and development are still largely unknown.^19^ Tyrosyl-tRNA Synthetase 2 (YARS2) is responsible for conjugating tyrosine to its cognate mt-tRNA for mitochondrial protein synthesis, supporting mitochondrial tRNA metabolisms.^20^ Variants in YARS2 have been reported to be associated with myopathy, lactic acidosis, and deafness.^21^ Although some other specific nuclear mitochondrial genes have been found to correlate with clinical outcomes of multiple tumors.^22,23^ Physiological functions of YARS2 in cancer progressions remains poorly understood. In the present study, we detected the expression of YARS2 in CRC tissues and further examined the role of YARS2 using stable knockdown cell lines.

## Results

### YARS2 was upregulated in CRC tissues

**Figure 1.**
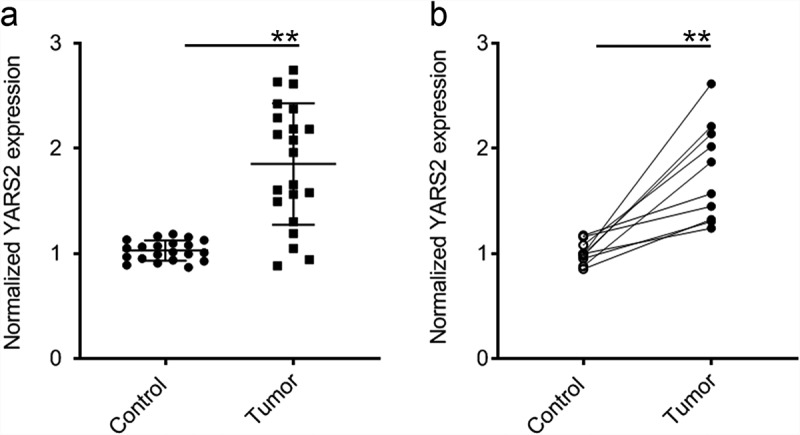
YARS2 was overexpressed in CRC tissues. (a) Overexpression of YARS2 mRNA in CRC tissues than normal tissues by RT-PCR analysis from local database in authors’ institute. (b) Detection of YARS2 mRNA expression in CRC tissues and adjacent non-tumor tissues collected from 10 patients with stage III colorectal adenocarcinoma. The expression levels of YARS2 were normalized to those of β-actin (**, *P* < .01), n = 10.

To evaluate whether YARS2 is involved in CRC progression, the expression profile of YARS2 in CRC and normal tissues was assessed. We first analyzed the mRNA expression of YARS2 in our local database (data not shown). The data showed that the mRNA expression of YARS2 was overexpressed in CRC tissues compared to non-tumor control tissues (*P* < .01) (Figure 1a). In addition, CRC tissues and adjacent non-tumor tissues were collected from 10 patients with stage III colorectal adenocarcinoma enrolled in the present study. The consistent results were observed that CRC tissues exhibited higher mRNA expression levels of YARS2 (*P* < .01) (Figure 1b).

### Knockdown of YARS2 inhibited CRC cell proliferation, migration, and sensitized to 5-FU

**Figure 2.**
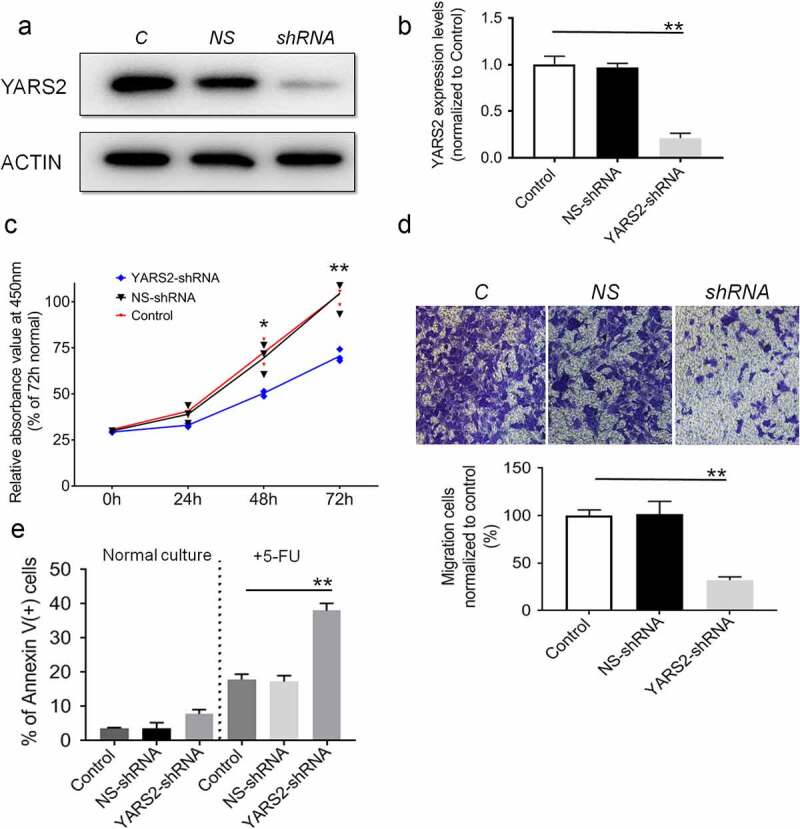
Knockdown of YARS2 inhibited CRC cell proliferation and migration, and sensitized cells to 5-FU treatment. (a) Western blot analysis of YARS2 protein in the lysates of either the untreated cells (C, control) or the cells transfected with a non-silencing shRNA (NS, scrambled sequence) or transfected with a specific YARS2 shRNA (shRNA). ACTIN, β-actin expression was used as a loading control. (b) The optical density was determined using NIH ImageJ software. The ratio of YARS2 expression to B-ACTIN expression was determined and normalized. (c) CCK8 assay results showing the effect of reduced expression of YARS2 on growth in SW620 cells compared to that of controls. (d) Transwell migration assay of SW620 cells for 24 hours. (e) Apoptotic ratios of SW620 cells before or after 5-FU treaments were determined by flow cytometry. Data are expressed as means ± SD. *, *P* < .05; **, *P* < .01, n = 3.

To explore the function of YARS2 in CRC cells, we knocked down YARS2 in the human CRC cell line SW620 via lentivirus-mediated shRNA. The knockdown efficiency was demonstrated by western blotting. As shown in Figure 2a, YARS2 in SW620 cells was stably knockdown, resulting in ~75% reduction of the protein levels (Figure 2b). Results of the CCK8 assay suggested that cell proliferation was attenuated in YARS2 knockdown SW620 cells (Figure 2c). Meanwhile, transwell migration analysis showed that the number of migrative cells was significantly decreased in YARS2 knockdown SW620 cells compared to both parental control and NS control cells (Figure 2d). Furthermore, three groups of SW620 cells were cultured in a growth medium with or without 1.5 μg/ml 5-FU (S1209, Selleck Chemicals, prepared by growth medium) for 2 days, which is a conventional anti-cancer chemotherapy drug for CRC. As shown in Figure 2e, YARS2 knockdown SW620 cells generally exhibited a little bit higher apoptotic ratio in normal culture compared to the two controls; following 5-FU treatment, the apoptosis was induced in approximately 17.9 ± 1.5% in parental control cells, 17.3 ± 1.8% in NS control cells, and the apoptosis ratios were significantly increased to 38.1 ± 2.1% in YARS2 knockdown SW620 cells (all P < .01). We also tested the role of YARS2 in another CRC cell line HT29. Similarly, the results showed that knockout of YARS2 in HT29 cell line resulted in decreased cell proliferation and significantly increased cell apoptosis induced by 5-FU. In addition, re-expression of YARS2 by a rescue experiment largely abolished these phenomenons (Supplementary Figure 1).

### YARS2 expression was associated with the level of mitochondrial tRNATyr and mitochondrial proteins in CRC cells

**Figure 3.**
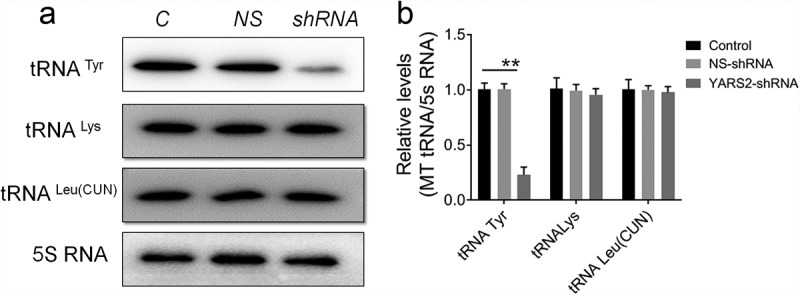
Northern-blot analysis of mitochondrial tRNA in three group of SW620 cells. (a) Equal amounts of total mtRNA samples from the three group of cells were adopted, electroblotted and hybridized with specific oligonucleotide probes for tRNA Tyr, tRNA Lys, tRNA Leu^26262626262423222121^, and 5S RNA, respectively. (b) Quantification of the tRNA levels were performed. The values are normalized to the control cells. Data are expressed as means ± SD. **, *P* < .01, n = 3.

**Figure 4.**
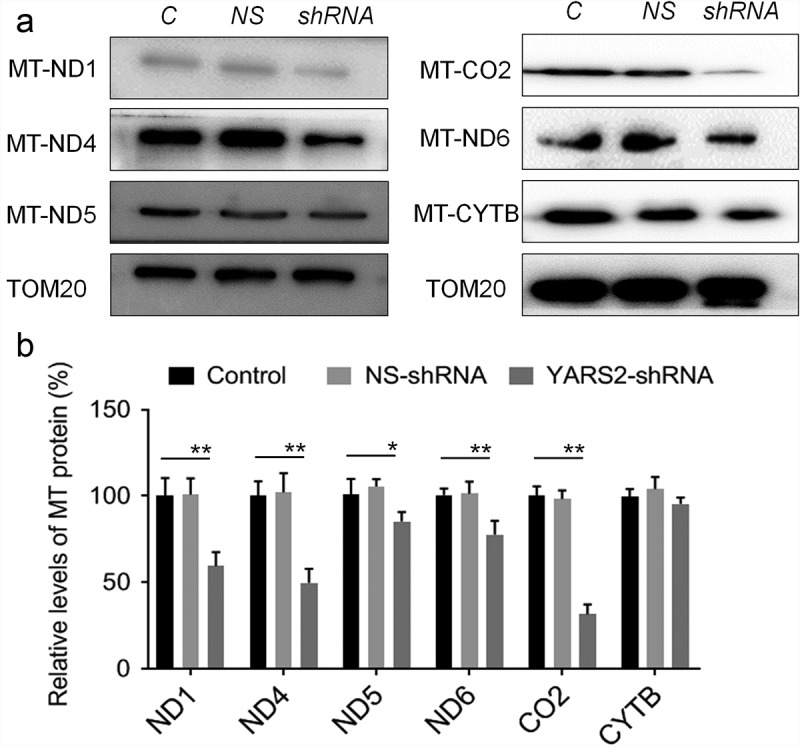
Determination of mitochondrial protein levels by Western blot assay. (a) Representative Western blot that hybridized with mitochondrial respiratory complex subunits of SW620 cells. TOM20 was adopted as loading control. MT-ND1, MT-ND4, MT-ND5 and MT-ND6 belong to subunit of complex I; MT-CO2 belongs to subunit of complex IV; MT-CYTB belongs to subunit of complex III. (b) Quantification of mitochondrial protein levels. The optical density was determined using NIH ImageJ software. The values are expressed as percentages of the average values for the control cells. Data are expressed as means ± SD. *, P < .05; **, P < .01, n = 3.

Given the involvement of YARS2 in mitochondrial function as determined by other research groups,^24,25^ we first investigated whether knockdown YARS2 in SW620 cells affects the steady-state level of tRNAs. As shown in Figure 3, the level of tRNATyr was significantly decreased in YARS2 knockdown cells (*P* < .01). Meanwhile, the average steady-state levels of tRNALys and tRNALeu^26^ were comparable in all three groups of cells, suggesting that the decreased effect on tRNATyr by YARS2 knockdown is specific. Then, we performed a western blot analysis to evaluate the protein levels of representative respiratory complex subunits in these SW620 cells. As shown in Figure 4, the expression levels of all tested proteins were comparable between parental control and NS control group, while the YARS2 knockdown cells exhibited markedly changed levels of MT-ND1 (subunit of complex I, ~45% reduction, P < .01), MT-ND4 (subunit of complex I, ~50% reduction, P < .01), MT-ND5 (subunit of complex I, ~15% reduction, P < .05), MT-ND6 (subunit of complex I, ~21% reduction, P < .01), MT-CO2 (subunit of complex IV, ~70% reduction, P < .01), and MT-CYTB (subunit of complex III, ~8% reduction, P > .05), respectively.

### Knockdown of YARS2 in CRC cells leads to defects in respiration

**Figure 5.**
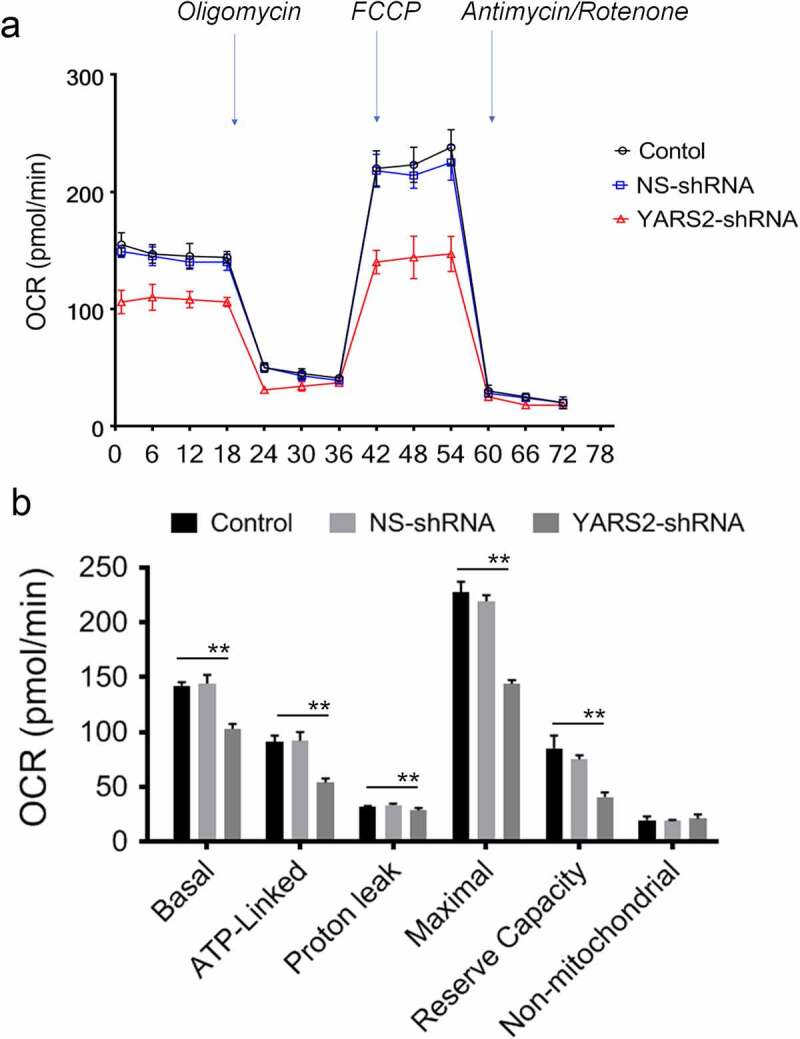
Seahorse Flux Analyzer bioenergetics analysis of YARS2 knockdown cells illustrates significant reduction in respiration. (a) Representative graph of the oxygen consumption rate (OCR). Oligomycin (1.5 μM), carbonyl cyanide p-(trifluoromethoxy)phenylhydrazone (FCCP) (0.5 μM), rotenone (1 μM) and antimycin A (1 μM) were added at indicated times to determine different parameters of mitochondrial functions. (b)The average basal OCR, ATP-linked production, proton leakage, maximal OCR, reserve respiratory capacity, as well as non-mitochondrial production were determined. Data are expressed as means ± SD. **, *P* < .01, n = 3.

**Figure 6.**
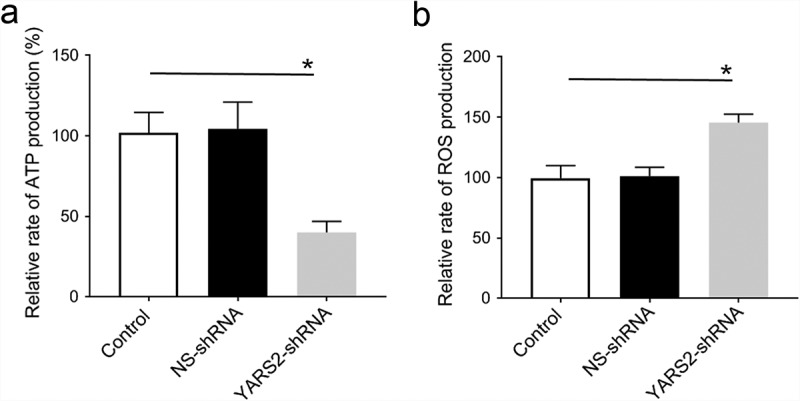
Determination of ATP and ROS levels in SW620 cells. (a) YARS2 knockdown cells exhibited significantly reduced mitochondrial ATP levels. (b) Relative levels of ROS production. Data are expressed as means ± SD. *, *P* < .05, n = 3.

To further evaluate the effect of YARS2 knockdown in CRC cells, the cellular oxygen consumption rates (OCR) were determined using a seahorse instrument. OCR was measured after the sequential addition of oligomycin, FCCP, rotenone, and antimycin. As shown in Figure 5, the OCR curves closely overlapped between the two control groups, indicating that there is no significant difference in OCR levels between the two controls. Compared to the controls, the basal OCR was reduced by 30% in YARS2 knockdown cells. More detailly, the ATP-linked OCR, proton leak OCR, maximal OCR, and reserve capacity in YARS2 knockdown cells was ~56%, ~90%, ~64%, and ~51% relative to the mean value measured in controls, respectively (all *P* < .01). No significant difference in non-mitochondrial OCR was observed. As expected, the total ATP generation was reduced in YARS2 knockdown cells (Figure 6a), while the reactive oxygen species (ROS) generation was increased (Figure 6b). Raised level of ROS has also been detected in HT29 cell line with knockout of YARS2, which is largely abolished by rescue experiment (Supplementary Figure 1).

### Knockdown of YARS2 in CRC cells leads to delayed in vivo progression in mice model

**Figure 7.**
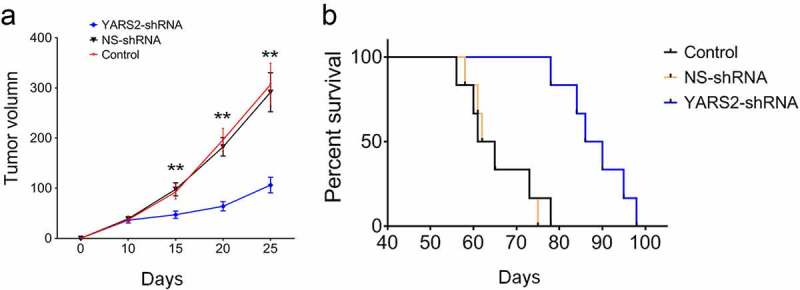
YARS2 knockdown SW620 cells showed reduced progression in mice model. (a) Total tumor volume at 10, 15,20, and 25 days after the implantation of CRC cells. (b) Kaplan-Meier plot of overall survival of pelvic recurrence model. Data are expressed as means ± SD. **, *P* < .01, n = 8.

To further validate the effect of YARS2 on CRC tumorigenic progression in vivo, three groups of SW620 cells were subcutaneously injected into the flanks of Balb/c nude mice. Stable knockdown of YARS2 was determined in SW620 cell-induced tumors by western blotting (Supplementary Figure 2). As shown in Figure 7a, tumor volume was determined for each mouse as described time points. The tumor size was significantly smaller in the YARS2 knockdown group by day 15 after cell implantation (*P* < .01), and the differences were enlarged along with the progress of the experiment. The median survival of mice in the parental control and NS control group was, respectively, 63.0 and 63.5 days, which was extended to 88.0 days in mice of the YARS2 knockdown group. These data suggest that depressed YARS2 inhibited CRC tumor growth in vivo.

## Discussion

Aminoacyl-tRNA synthetases (ARSs) are a family of evolutionarily conserved enzymes, which play a vital role in protein synthesis. These enzymes catalyze the ligation of specific amino acids to the corresponding tRNAs, known as aminoacylation. YARS2, which is encoded by the nuclear genome and functions in mitochondria, catalyzes the binding of tyrosine to the cognate mitochondrial tRNAs.^24^ According to Pubmed and the Human Genome Mutation Database, pathogenic YARS2 mutations have been linked to myopathy with lactic acidosis and sideroblastic anemia type 2 (MLASA2),^27^ Cardiomyopathy,^28^ and Sideroblastic anemia with myopathy.^29^ In these studies, the patients all carried pathogenic mutations which leads to abnormal mitochondrial function in various tissues, especially skeletal muscles, neurons, etc., indicating that stable expression of YARS2 is essential for cell function and tissue development.

Translation dysregulation has been demonstrated to contribute to CRC development and progression, as well as chemoresistance;^30,31^ however, relatively little is known about the changes that occur to the translational system in CRC. In the present study, we for the first time reported that YARS2 was overexpressed in CRC tissues compared to either healthy colorectal tissue or the correlated adjacent tissues. Then, we adopted human colon cancer cell-line SW620 as a cultured model and knocked down YARS2 expression. With a reduced expression of YARS2, SW620 cells exhibited a decreased steady-state level of tRNA^Tyr^, which led to reduced levels of mitochondrial protein expression. These data confirm the role of YARS2 in CRC cells. Moreover, YARS2 knockdown SW620 cells showed a reduced level of ATP which may be responsible for the inhibited cell proliferation and tumor growth. Increased ROS generation by YARS2 knockdown might be responsible for the enhancing sensitivity to 5-FU of SW620 cells, as determined by the result that antioxidant N-acetylcysteine treatment significantly reverse increased apoptosis ratio induced by 5-FU (Supplementary Figure 3).

CRC persists as one of the most prevalent and deadly tumor types in both men and women worldwide. Despite advances in systemic therapy, the 5-year survival rate is still a mere 12.5%; resistance to chemotherapy remains one of the greatest challenges. Of note, we also found in this study that inhibition of YARS2 is associated with increased reactive oxygen species levels which sensitize these cells to 5-FU treatment. Consequently, YARS2 might be a potential target to overcome resistance in CRC. However, future studies are warranted to explore the underlying mechanism of upregulated YARS2 on CRC cells.

## Materials and methods

### Clinical samples

The present study was approved by the Ethics Committee of Zhejiang Provincial People’s Hospital in Hangzhou, China. Written informed consents were obtained from all patients for samples collection.

### Cell lines and culture conditions

The human colon cancer cell-line SW620 was purchased from the American Type Culture Collection (ATCC, USA). To stably knock down YARS2 in SW620 cells, the short hairpin RNA (shRNA, purchased from Santa Cruz Biotechnology) containing a hairpin loop was synthesized and inserted into the pLKO.1-puro vector (Addgene, #8453), and selected by 2 μg/mL puromycin (Sigma-Aldrich). A scrambled shRNA was used as the negative control. All the CRC cells were cultured in a humidified atmosphere of 5% CO_2_ and 95% air with DMEM (Sigma-Aldrich) supplemented with 10% fetal bovine serum (FBS, Gibco).

### RNA isolation and quantitative real-time PCR (qRT-PCR)

Total RNAs were extracted using TRIzol reagent (Takara Bio, Shanghai, China) from CRC and normal intestinal tissues according to the manufacturer’s protocol, and reverse transcribed into cDNA using the PrimeScript™ RT Reagent Kit (Takara Bio). qRT-PCR was performed to determine the relative mRNA levels of YARS2 with specific primers purchased from Sinobiological (Cat: HP103463 for human YARS2; Cat: HP100001 for human beta-Actin). All PCRs were performed in a total volume of 20 µL using an SYBR Premix Ex Taq Kit (Takara Bio). The amplification conditions for qRT-PCR were as follows: 95°C for 30 s, followed by 45 cycles of 95°C for 5 s and 60°C for 20 s, and 95°C for 15 s. Relative expression levels were calculated using the 2-ΔΔCT method. Each sample was evaluated in triplicate and measurements were repeated independently at least three times.

### Cell growth analysis

This assay was performed using a CCK8 cell viability kit (Yeasen, China) according to the introduced protocol. Briefly, CRC cells growing in log-phase were trypsinized, resuspended, and seeded at a density of 2 × 10^3^ cells/well in 96-well plates. At the indicated time point, 10 μl of CCK8 was added to each well for 1 h at 37°C. Then, the absorbance was measured at 450 nm by Synergy H1 (BioTek, China).

### Migration assay

For cell migration assay, cells were harvested and re-suspended in DMEM with 1% FBS. Then, 2 × 10^4^ cells were seeded into the upper chamber of transwell filter chambers (Corning, 4.5-μm pores), and the lower chambers were filled with 2 mL DMEM containing 10% FBS. After 18 hours of incubation, cells that migrated into the reverse side of the transwell membrane were fixed with methanol, stained with Crystal Violet, and then counted under an inverted light microscope.

### Apoptosis analysis by flow cytometry

Apoptosis of cells after various treatments was measured using Annexin V-propidium iodide binding assay (BD Pharmingen) according to the manufacturer’s instructions. The fluorescence intensity was determined using an ACEA NovoCyte cytometer. The numbers of apoptotic cells were expressed as percentages of Annexin V-posotive cells. All experiments were performed in triplicates.

### Western blotting analysis

Western blotting analysis was performed as detailed previously.^32^ Antibodies used in this study include anti-YARS2 (ab228957, at a dilution of 1:1000), anti-MT-ND1 (ab181848, at a dilution of 1:2000), anti-MT-ND5 (ab230509, at a dilution of 1:1000), anti-MT-CO2 (ab79393, at a dilution of 1:5000), and anti-TOM20 (ab186735, at a dilution of 1:2000) from Abcam, anti-MT-ND6 (PA5-43532, at a dilution of 1:1000) and anti-MT-ND4 (PA5-97298, at a dilution of 1:1000) from ThermoFisher, anti- MT-CYTB (55090-1-AP, at a dilution of 1:1000) from Proteintech, anti-β-actin (AF5003, at a dilution of 1:2000) from Beyotime Institute of Biotechnology, and HRP-conjugated anti-rabbit secondary antibody (A0208, at a dilution of 1:1000) from Beyotime Institute of Biotechnology. Immunoreactive proteins were visualized using a chemiluminescent immunodetection system (ChemiDoc XRS). Image J was employed to analyze the grayscale values obtained by western blotting.

### Northern blotting for mitochondrial tRNA analysis

Total mitochondrial RNAs were isolated using RiboPure™ RNA kit (Invitrogen). For northern blotting, 2 μg of total mitochondrial RNA were first electrophoresed in 10% polyacrylamide/7 M urea gel, then electroblotted onto a positively charged nylon membrane for the hybridization analysis with non-radioactive digoxin (DIG)-labeled oligodeoxynucleotide probes (Roche). Image J was employed to analyze the grayscale values for quantification.

### Measurement of oxidative phosphorylation (OCR), adenosine triphosphate (ATP), and reactive oxygen species (ROS)

Cellular OCR alternations were determined with the Seahorse XF96 Flux Analyzer (Seahorse, Agilent) in real-time according to the manufacturer’s instructions. In brief, plate-based live cells were assayed for monitor OCR in real time. The modulators included in this assay are Oligomycin, Carbonyl cyanide-4 (trifluoromethoxy) phenylhydrazone (FCCP), Rotenone, and Antimycin. Oligomycin is used as a complex V inhibitor to assess ATP production levels. FCCP is used as a mitochondrial uncoupler to assess maximal respiration, and rotenone or antimycin treatment is used as amitochondrial complex I uncoupler to assess non-mitochondrial respiration. The results of different groups were normalized by cell numbers, which were automatically analyzed by Seahorse XF96 Flux Analyzer. The cellular ATP and ROS levels were, respectively, measured by standard assay Kits purchased from Beyotime according to the manufacturer’s instructions.

### Animal models

Eight-week-old female Balb/c nude mice were purchased from the Beijing Vital River Laboratory Animal Technology Co. All animal procedures have been performed in accordance with protocols approved by the local Institutional Animal Care and Use Committee. The mice were housed in filter-top cages in a closed, environment-controlled colony and were provided sterile food and water. For mouse xenograft models, SW620 cells (5 × 10^6^/0.1 mL Matrigel, Sigma-Aldrich) were injected subcutaneously into the lower right flank of female nude mice. Animal health and tumor growth were monitored daily. Tumor volumes were estimated by external caliper, and the greatest longitudinal diameter (length) and the greatest transverse diameter (width) were determined. Tumor volume was calculated by the modified ellipsoidal formula: V = ½ (Length × Width^2^). In order to assess interobserver variation, each tumor was measured by three independent observers.

### Statistical analysis

All data are presented as means ± standard deviation (SD) of the results of at least three independent studies. Statistical differences between the two groups were evaluated by Student’s t-tests. Statistical significance was defined as P < .05.

## Supplementary Material

Supplemental Material

## Data Availability

The data of this study are available from the corresponding author upon reasonable request.

## References

[1] Ganesh K, Stadler ZK, Cercek A, Mendelsohn RB, Shia J, Segal NH, Diaz LA. Immunotherapy in colorectal cancer: rationale, challenges and potential. Nat Rev Gastroenterol Hepatol. 2019;16(6):361–375. doi:10.1038/s41575-019-0126-x. 30886395 PMC7295073

[2] Dekker E, Rex DK. Advances in CRC prevention: screening and surveillance. Gastroenterology. 2018;154(7):1970–1984. doi:10.1053/j.gastro.2018.01.069. 29454795

[3] Choi CH, Rutter MD, Askari A, Lee GH, Warusavitarne J, Moorghen M, Thomas-Gibson S, Saunders BP, Graham TA, Hart AL. Forty-year analysis of colonoscopic surveillance program for neoplasia in ulcerative colitis: an updated overview. Am J Gastroenterol. 2015;110(7):1022–1034. doi:10.1038/ajg.2015.65. 25823771 PMC4517513

[4] Siegel RL, Miller KD, Fuchs HE, Jemal A. Cancer Statistics, 2021. Ca Cancer J Clin. 2021;71(1):7–33. doi:10.3322/caac.21654. 33433946

[5] Wong SH, Yu J. Gut microbiota in colorectal cancer: mechanisms of action and clinical applications. Nat Rev Gastroenterol Hepatol. 2019;16(11):690–704. doi:10.1038/s41575-019-0209-8. 31554963

[6] Cheng Y, Ling Z, Li L. The intestinal microbiota and colorectal cancer. Front Immunol. 2020;11:615056. doi:10.3389/fimmu.2020.615056. 33329610 PMC7734048

[7] Xu R, Wang W, Zhu B, Lin X, Ma D, Zhu L, Zhao Q, Nie Y, Cai X, Li Q, Fang W. Disease characteristics and treatment patterns of Chinese patients with metastatic colorectal cancer: a retrospective study using medical records from China. BMC Cancer. 2020;20(1):131. DOI:10.1186/s12885-020-6557-5 32070312 PMC7029588

[8] Wei EK, Giovannucci E, Wu K, Rosner B, Fuchs CS, Willett WC, Colditz GA. Comparison of risk factors for colon and rectal cancer. Int J Cancer. 2004;108(3):433–442. doi:10.1002/ijc.11540. 14648711 PMC2903217

[9] Lynch HT, Smyrk TC, Watson P, Lanspa SJ, Lynch JF, Lynch PM, Cavalieri RJ, Boland CR. Genetics, natural history, tumor spectrum, and pathology of hereditary nonpolyposis colorectal cancer: an updated review. Gastroenterology. 1993;104(5):1535–1549. doi:10.1016/0016-5085(93)90368-m. 8482467

[10] Pira G, Uva P, Scanu AM, Rocca PC, Murgia L, Uleri E, Piu C, Porcu A, Carru C, Manca A. Landscape of transcriptome variations uncovering known and novel driver events in colorectal carcinoma. Sci Rep. 2020;10(1):432. doi:10.1038/s41598-019-57311-z. 31949199 PMC6965099

[11] Xu H, Wang C, Song H, Xu Y, Ji G. RNA-Seq profiling of circular RNAs in human colorectal Cancer liver metastasis and the potential biomarkers. Mol Cancer. 2019;18(1):8. doi:10.1186/s12943-018-0932-8. 30630466 PMC6327571

[12] Chekulayev V, Mado K, Shevchuk I, Koit A, Kaldma A, Klepinin A, Timohhina N, Tepp K, Kandashvili M, Ounpuu L, et al. Metabolic remodeling in human colorectal cancer and surrounding tissues: alterations in regulation of mitochondrial respiration and metabolic fluxes. Biochem Biophy Rep. 2015;4:111–125. doi:10.1016/j.bbrep.2015.08.020. PMC5668899 29124194

[13] Chang L, Fang S, Gu W. The molecular mechanism of metabolic remodeling in lung cancer. J Cancer. 2020;11(6):1403–1411. doi:10.7150/jca.31406. 32047547 PMC6995370

[14] Jia D, Lu M, Jung KH, Park JH, Yu L, Onuchic JN, Kaipparettu BA, Levine H. Elucidating cancer metabolic plasticity by coupling gene regulation with metabolic pathways. J Proc Natl Acad Sci. 2019;116(9):3909–3918. doi:10.1073/pnas.1816391116. PMC6397570 30733294

[15] Neitzel C, Demuth P, Wittmann S, Fahrer J. Targeting altered energy metabolism in colorectal cancer: oncogenic reprogramming, the central role of the TCA cycle and therapeutic opportunities. Cancers. 2020;12(7):1731. doi:10.3390/cancers12071731. PMC7408264 32610612

[16] Xiao Z, Dai Z, Locasale JW. Metabolic landscape of the tumor microenvironment at single cell resolution. Nat Commun. 2019;10(1):3763. doi:10.1038/s41467-019-11738-0. 31434891 PMC6704063

[17] Sun X, Zhan L, Chen Y, Wang G, He L, Wang Q, Zhou F, Yang F, Wu J, Wu Y. Increased mtDNA copy number promotes cancer progression by enhancing mitochondrial oxidative phosphorylation in microsatellite-stable colorectal cancer. Signal Transduct Target Ther. 2018;3(1):8. doi:10.1038/s41392-018-0011-z. 29610678 PMC5878831

[18] Wei -T-T, Lin Y-T, Tang S-P, Luo C-K, Tsai C-T, Shun C-T, Chen -C-C. Metabolic targeting of HIF-1α potentiates the therapeutic efficacy of oxaliplatin in colorectal cancer. Oncogene. 2020;39(2):414–427. doi:10.1038/s41388-019-0999-8. 31477841

[19] La Vecchia S, Sebastián C. Metabolic pathways regulating colorectal cancer initiation and progression. Semin Cell Dev Biol. 2020;98:63–70. doi:10.1016/j.semcdb.2019.05.018. 31129171

[20] Bonnefond L, Fender A, Rudinger-Thirion J, Giegé R, Florentz C, Sissler M. Toward the full set of human mitochondrial aminoacyl-tRNA synthetases: characterization of AspRS and TyrRS. Biochemistry. 2005;44(12):4805–4816. doi:10.1021/bi047527z. 15779907

[21] Riley LG, Heeney MM, Rudinger-Thirion J, Frugier M, Campagna DR, Zhou R, Hale GA, Hilliard LM, Kaplan JA, Kwiatkowski JL. The phenotypic spectrum of germline YARS2 variants: from isolated sideroblastic anemia to mitochondrial myopathy, lactic acidosis and sideroblastic anemia 2. Haematologica. 2018;103(12):2008. doi:10.3324/haematol.2017.182659. 30026338 PMC6269294

[22] Su CY, Chang YC, Yang CJ, Huang MS, Hsiao M. The opposite prognostic effect of NDUFS1 and NDUFS8 in lung cancer reflects the oncojanus role of mitochondrial complex I. Sci Rep. 2016;6(1):1–8. doi:10.1038/srep31357. 27516145 PMC4981865

[23] Zhang X, Dong W, Zhang J, Liu W, Yin J, Shi D, Ma W. A novel mitochondrial-related nuclear gene signature predicts overall survival of lung adenocarcinoma patients. Front Cell Dev Biol. 2021;9:740487. doi:10.3389/fcell.2021.740487. 34760888 PMC8573348

[24] Riley LG, Cooper S, Hickey P, Rudinger-Thirion J, McKenzie M, Compton A, Lim SC, Thorburn D, Ryan MT, Giegé R. Mutation of the mitochondrial tyrosyl-tRNA synthetase gene, YARS2, causes myopathy, lactic acidosis, and sideroblastic anemia–MLASA syndrome. Am J Hum Genet. 2010;87(1):52–59. doi:10.1016/j.ajhg.2010.06.001. 20598274 PMC2896778

[25] Carreno-Gago L, Juarez-Flores DL, Grau JM, Ramon J, Lozano E, Vila-Julia F, Martí R, Garrabou G, Garcia-Arumí E. Two novel variants in YARS2 gene are responsible for an extended MLASA phenotype with pancreatic insufficiency. J Clin Med. 2021;10(16):3471. doi:10.3390/jcm10163471. 34441767 PMC8397107

[26] Douillard J, Cunningham D, Roth A, Navarro M, James R, Karasek P, Jandik P, Iveson T, Carmichael J, Alakl M. Irinotecan combined with fluorouracil compared with fluorouracil alone as first-line treatment for metastatic colorectal cancer: a multicentre randomised trial. Lancet. 2000;355(9209):1041–1047. doi:10.1016/S0140-6736(00)02034-1. 10744089

[27] Sommerville EW, Ng YS, Alston CL, Dallabona C, Gilberti M, He L, Knowles C, Chin SL, Schaefer AM, Falkous G, et al. Clinical features, molecular heterogeneity, and prognostic implications in YARS2 -related mitochondrial myopathy. JAMA Neurol. 2017;74(6):686–694. DOI:10.1001/jamaneurol.2016.4357 28395030 PMC5822212

[28] Gurtner C, Hug P, Kleiter M, Köhler K, Dietschi E, Jagannathan V, Leeb T. YARS2 missense variant in belgian shepherd dogs with cardiomyopathy and juvenile mortality. Genes. 2020;11(3):313. doi:10.3390/genes11030313. PMC7140874 32183361

[29] Smith F, Hopton S, Dallabona C, Gilberti M, Falkous G, Norwood F, Donnini C, Gorman GS, Clark B, Taylor RW. Sideroblastic anemia with myopathy secondary to novel, pathogenic missense variants in the YARS2 gene. Haematologica. 2018;103(12):e564–e566. doi:10.3324/haematol.2018.194464. 29976739 PMC6269300

[30] Schmidt S, Denk S, Wiegering A. Targeting protein synthesis in colorectal cancer. Cancers. 2020;12(5):1298. doi:10.3390/cancers12051298. PMC7281195 32455578

[31] Mao Z, Zhao H, Qin Y, Wei J, Sun J, Zhang W, Kang Y. Post-Transcriptional dysregulation of microRNA and alternative polyadenylation in colorectal cancer. Front Genet. 2020;11(64). doi:10.3389/fgene.2020.00064. PMC7047281 32153636

[32] Fang QX, Zheng XC, Zhao HJ. L1CAM is involved in lymph node metastasis via ERK1/2 signaling in colorectal cancer. Am J Transl Res. 2020;12(3):837–846. 32269716 PMC7137048

