# Supplementary figures and images for: Targeting mitochondrial tyrosyl-tRNA synthetase YARS2 suppresses colorectal cancer progression
Source: Cancer Biol Ther. 2022 Sep 25;23(1):1–8. doi: 10.1080/15384047.2022.2127603 (PMC9518999; doi:10.1080/15384047.2022.2127603)

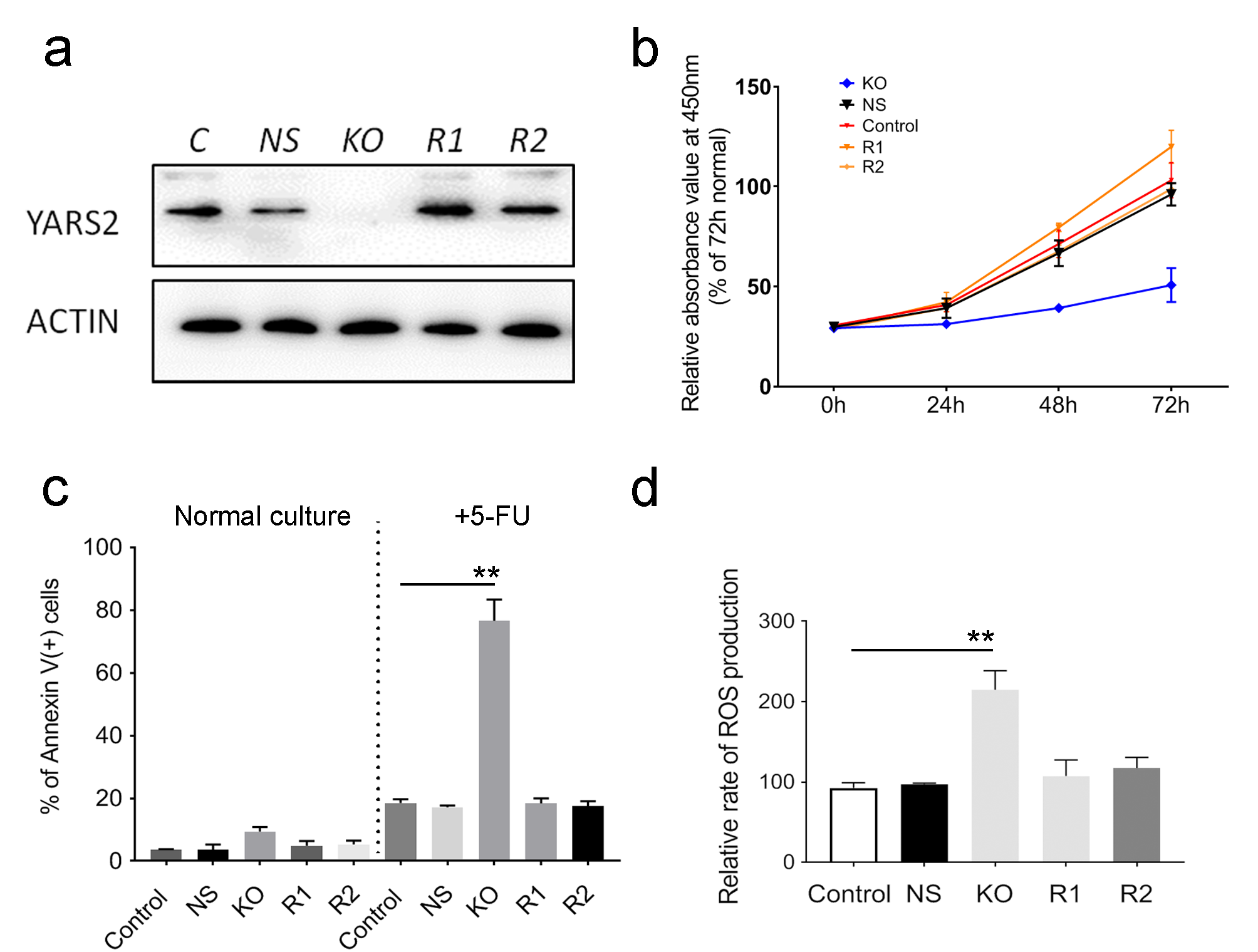

Supplement: Supplemental Material [file KCBT_A_2127603_SM9163.zip › supplementary Figure1.tif]

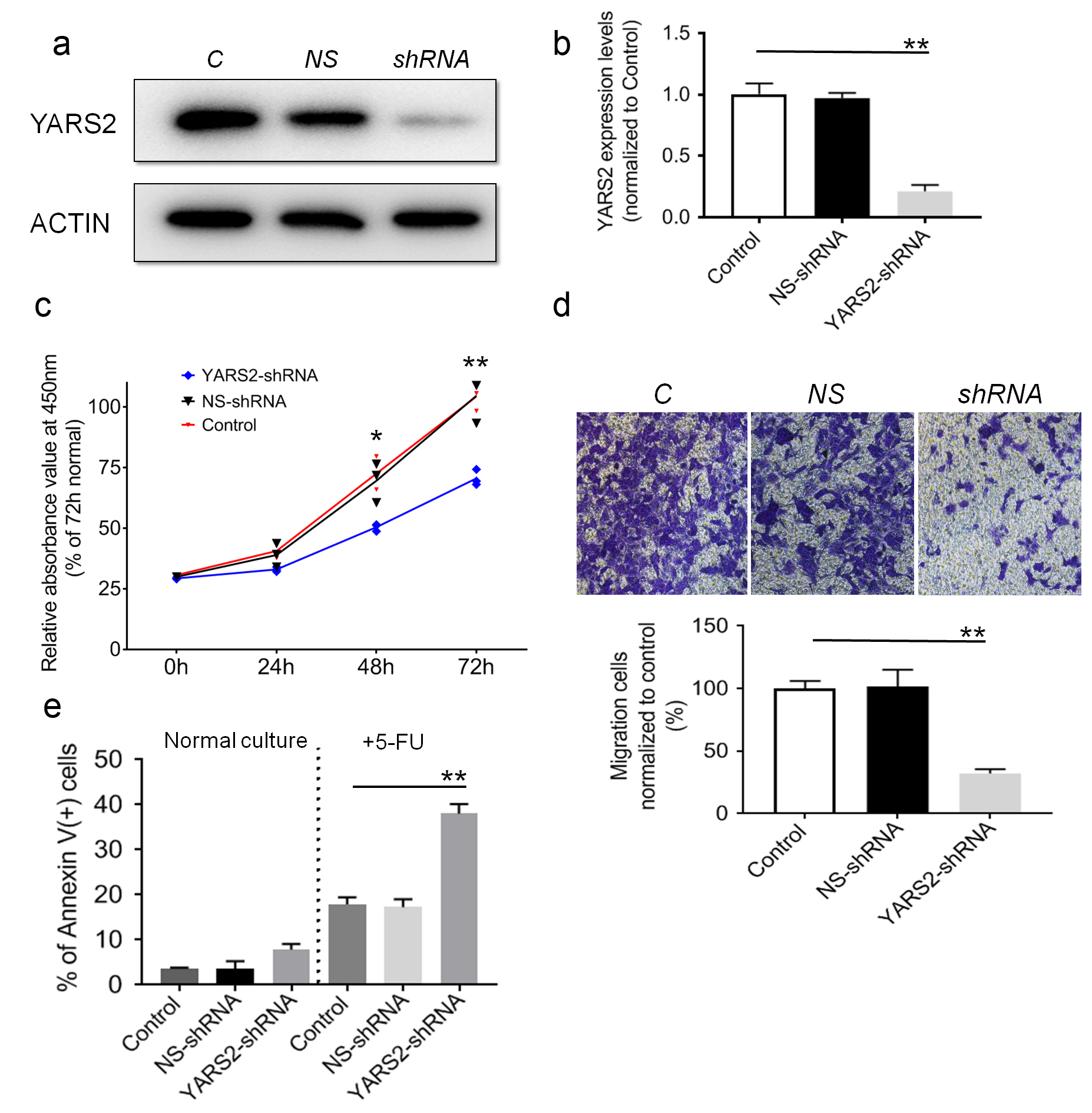

Supplement: Supplemental Material [file KCBT_A_2127603_SM9163.zip › supplementary Figure2.tif]

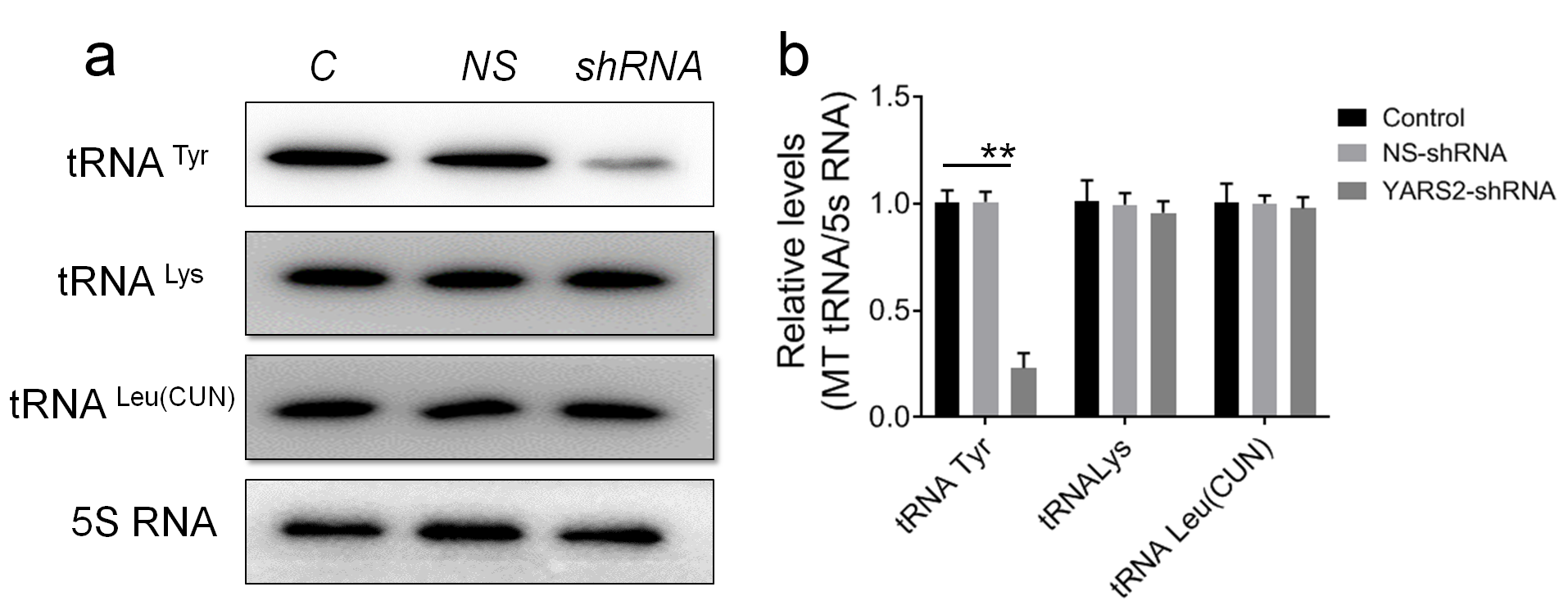

Supplement: Supplemental Material [file KCBT_A_2127603_SM9163.zip › supplementary Figure3.tif]
